# Methodology for the analysis of rare genetic variation in genome-wide association and re-sequencing studies of complex human traits

**DOI:** 10.1093/bfgp/elu012

**Published:** 2014-06-10

**Authors:** Loukas Moutsianas, Andrew P. Morris

**Keywords:** rare variant, burden test, dispersion test, statistical methodology, genome-wide association, whole-genome and whole-exome re-sequencing

## Abstract

Genome-wide association studies have been successful in identifying common variants that impact complex human traits and diseases. However, despite this success, the joint effects of these variants explain only a small proportion of the genetic variance in these phenotypes, leading to speculation that rare genetic variation might account for much of the ‘missing heritability’. Consequently, there has been an exciting period of research and development into the methodology for the analysis of rare genetic variants, typically by considering their joint effects on complex traits within the same functional unit or genomic region. In this review, we describe a general framework for modelling the joint effects of rare genetic variants on complex traits in association studies of unrelated individuals. We summarise a range of widely used association tests that have been developed from this model and provide an overview of the relative performance of these approaches from published simulation studies.

## INTRODUCTION

Genome-wide association studies (GWAS) have been extremely successful in identifying loci contributing to a wide range of complex human traits and diseases [1]. However, association signals in these loci are typically characterised by common lead single nucleotide polymorphisms (SNPs), each of modest effect, which when considered together account for only a small proportion of the genetic variance of the trait [2]. For example, the 180 reported loci for human height in the general population together explain no more than 10% of the genetic variance of the trait [3], whilst the joint effects of lead SNPs at 63 established loci for type 2 diabetes account for less than 6% of the familial aggregation of the disease [4]. Although there may be many additional common SNPs with effects on complex traits that are too modest to have been discovered through current GWAS efforts [5], it seems unlikely that the ‘common disease, common variant’ paradigm will be all encompassing. Consequently, there has been much recent debate as to the role that rare genetic variation, often defined to have minor allele frequency (MAF) of less than 1%, might play in explaining the ‘missing heritability’ of complex human traits [6, 7].

Rare genetic variants are likely to have arisen from mutation events in the last 20 generations, and thus are more likely than common SNPs to be ethnic specific or polymorphic in just one population [8]. They are also expected to have larger effects on complex traits than common variants because they will not have been subject to purifying selection after the recent expansion of the human population [9]. However, because of the low MAF, these effects are unlikely to be sufficiently large to be detected with the usual single SNP association tests used in the analysis of GWAS. Furthermore, traditional genotyping platforms used in GWAS have primarily been designed to capture common SNPs, taking advantage of the structure of linkage disequilibrium throughout the genome, but offer only poor coverage of rare genetic variation [10].

The most comprehensive approach to assaying rare genetic variation is through large-scale re-sequencing studies [11]. With considerable improvements in the throughput and efficiency of these technologies, whole-genome or whole-exome re-sequencing in large sample sizes is increasingly becoming a realistic financial undertaking for many research groups. Furthermore, high-density reference panels from the 1000 Genomes Project Consortium, derived from large-scale re-sequencing efforts in multiple populations, provide a comprehensive catalogue of genetic variation with MAF as low as 0.5% across ethnic groups, as well as many rarer variants [12, 13]. Such reference panels could be used to select rare variants for inclusion on custom-designed arrays, potentially with priority given to those with likely functional consequences, such as the Illumina Infinium HumanExome BeadChip, enabling cost-effective genotyping in the large sample sizes required for complex trait association studies. Furthermore, if samples have already been assayed with traditional GWAS arrays, imputation techniques can make use of this common SNP scaffold to predict genotypes at variants, including those of lower frequency, that are present in the higher density reference panel, incurring no additional cost, other than computation [14].

With the increasing availability of high-quality data from large-scale re-sequencing, genotyping and imputation studies of complex human traits, there has been an exciting period of development of statistical methodology for the analysis of rare genetic variation from this ‘next generation’ of GWAS. These methods have primarily focused on the analysis of rare variants within the same ‘functional unit’ (exon, gene or pathway) or genomic region, increasing power to detect association over single SNP approaches by considering their joint effects on complex traits. In this review, we describe a general framework for modelling the joint effects of rare genetic variants on complex traits in association studies of unrelated individuals. We summarise a range of widely used association tests that have been developed from this model and provide an overview of the relative performance of these approaches from published simulation studies.

## METHODOLOGY FOR THE ANALYSIS OF RARE GENETIC VARIATION

Consider a sample of unrelated individuals who have been typed for rare variants within some functional unit or genomic region. Within a generalised linear modelling (GLM) framework, we can model the phenotype, *y_i_*, of the *i*th individual as

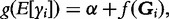



where *g*(.) is the link function. In this expression, *f*(.) is some function on the genotypes, ***G****_i_*, of the *i*th individual, typically coded as *G_ij_* = {0, 1, or 2} according to the number of minor alleles they carry at the *j*th variant. In an imputed GWAS, *G_ij_* is most often replaced by the expected genotype, *E*[*G_ij_*], under a dosage model. Specifically,

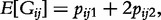



where *p_ij_*_1_ and *p_ij_*_2_ denote the imputed probabilities that the *i*th individual carries heterozygous and rare homozygous genotypes, respectively, at the *j*th variant. The properties of the rare variant association test are then determined by the form of the function *f*(.), as described in detail below.

Most rare variant statistical methodologies have been developed for quantitative traits (identity link function) or dichotomous phenotypes (logistic link function). However, the GLM can also incorporate more complex phenotypes including categorical responses and ‘time to event’ outcomes. Furthermore, the flexibility of the GLM framework facilitates incorporation of covariates to allow for adjustment for confounders, including non-genetic risk factors and indicators of population structure.

### Burden tests

Burden tests of association have been developed by modelling the effect of accumulations of minor alleles at rare variants, referred to as the ‘mutational load’, within some functional unit or genomic region. Under this model, *f*(.) is a simple linear function of the genotypes, **G**, given by

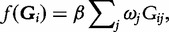



where *β* denotes the effect on the trait (log-odds ratio for a dichotomous phenotype) of each copy of a minor allele at rare variants within the functional unit or genomic region, and *ω_j_* ∊ [0,1] corresponds to the weight given to the *j*th rare variant. Consequently, each rare variant has the same direction, but not necessarily the same magnitude, of effect on the phenotype.

The simplest approach is to assume ‘unit weighting’, where *ω_j_* is an indicator variable, such that *ω_j_* = 1 if the *j*th rare variant is to be included in the analysis, and *ω_j_* = 0 otherwise. This ‘masking’ scheme may reflect annotation and/or frequency, so that only coding or non-synonymous variants are included in the analysis, for example, for some pre-specified MAF threshold. Such an approach has been implemented in GRANVIL [15, 16], where

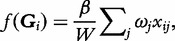



and 
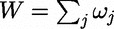
. Furthermore, in GRANVIL, genotypes are recoded under a dominant model such that *x_ij_* = 1 if *G_ij_* > 0, and *x_ij_* = 0 otherwise, or by *x_ij_* = *p_ij__1_* + *p_ij__2_* for an imputed GWAS, because the rare homozygous genotype is so infrequent. GRANVIL then uses a likelihood ratio test of the null hypothesis of no association, *β* = 0, of the trait with rare variants in the functional unit or genomic region.

An alternative approach to modelling the mutational load of a functional unit or genomic region is to ‘collapse’ rare variants into a ‘super-allele’ such that

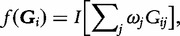



where 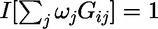 if 
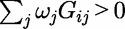
, and 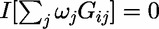, otherwise. This collapsing technique has been implemented in a Fisher’s exact test for a 2 × 2 contingency table for dichotomous phenotypes in CAST [17] and CCRaVAT [18], and in an ANOVA framework for quantitative traits in QuTie [18]. The combined multivariate and collapsing method extends this approach to allow for simultaneous analysis of multiple super-alleles in a regression framework [19]. In this context, each super-allele might correspond to alternative non-overlapping masking schemes for the same set of variants, for example, different MAF thresholds and/or annotation categories, or to variants in different functional units or genomic regions.

One of the disadvantages of the unit-weighting scheme described above is that a MAF threshold for inclusion of rare variants in the analysis must be specified in advance. Setting the MAF threshold too low might exclude important causal variants from the burden test, thereby reducing power. However, on the other hand, setting the MAF threshold too high might result in inclusion of many non-causal variants in calculating the mutational load, again resulting in a decrease in power. To overcome this problem, the variable threshold method considers multiple masking schemes for the same set of variants in a given functional unit or genomic region on the basis of MAF [20]. This approach has been motivated by the concept that there is some unknown ‘optimal’ MAF below which variants are most likely to have a direct impact on complex traits. Consequently, a test of association of the trait with the super-allele is performed at multiple MAF thresholds, with significance assessed by means of permutation.

Under the unit-weighting model, all rare variants included in the masking scheme are assumed to have the same magnitude of effect on the phenotype, as well as the same direction. As an alternative, the Madsen and Browning weighting scheme [21] allows lower-frequency variants to have a greater impact on the phenotype than on those that are more common, such that

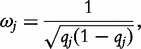



where *q_j_* is the MAF of the *j*th variant. The weighted sum statistic for dichotomous disease phenotypes makes use of this weighting scheme, based on the MAF in controls, to rank individuals according to their mutational load 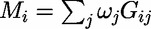 in the functional unit or genomic region [21]. A Wilcoxon test with permutation is then used to evaluate the significance of association by comparing ranks in cases and controls. The cumulative minor allele test provides a unified framework, allowing for general weighting schemes, taking account of both MAF and annotation [22].

### Generalised burden tests

As described above, an implicit underlying assumption of burden tests is that of the same direction of effect on phenotype of all rare variants in the same functional unit or genomic region. To remove this restrictive assumption, Han and Pan [23] proposed the data adaptive sum test (aSUM), which redefines the weighting scheme as *ω_j_* = 1 if 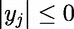, and *ω_j_* = −1 otherwise, where *γ_j_* is an estimate of the effect of the minor allele for the *j*th variant on the phenotype from a single variant GLM, for example. Under this model, a score test of the null hypothesis of no association between the trait and rare variants in the functional unit or genomic region is given by

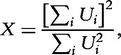



where

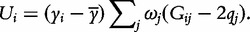



In this expression, 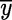 is the mean trait across individuals, and *q_j_* is the MAF of the *j*th rare variant. However, in aSUM, the same data are used to determine the weights, *ω_j_*, and to perform the score test of association. Consequently, the significance of the association is determined by permuting phenotypes, and recalculating weights and the test statistic across replicates. As an alternative, the data can be split, with weights derived in a training set and association testing undertaken in the remainder of samples, eliminating the need for computationally demanding permutations [24].

The aSUM test was extended by Hoffman *et al.* [25] by means of a ‘step-up’ approach, which considers a more general weighting scheme, defined by *ω_j_* = *a_j_δ_j_v_j_*. In this expression, *δ_j_* depends on the direction of the effect of the *j*th variant, as in the aSUM test. For dichotomous disease phenotypes, *δ_j_* = −1 if the *j*th variant is more prevalent in controls than cases, and *δ_j_* = 1 otherwise, whilst for quantitative traits, *δ_j_* denotes the sign of the correlation coefficient with the minor allele at the *j*th variant. The quantity *a_j_* is a continuous weighting function for the *j*th variant which could, for example, allow for Madsen and Browning weights [21]. Finally, *v_j_* is an indicator variable representing the masking scheme, taking the value *v_j_* = 1 if the *j*th variant is included in the analysis, and *v_j_* = 0 otherwise. This indicator variable could be defined to reflect annotation and/or frequency. In the ‘step-up’ approach, forward selection is used to identify the subset of variants that maximise the evidence of association with the trait. At each stage of this iterative process, the variant that maximises the increase in the score statistic, *X*, is selected in the model and continued until no further variants increase the evidence of association. The significance of the association is then determined by permuting phenotypes and repeating the model selection in each replicate.

### Dispersion tests

The aSUM and ‘step-up’ methods alleviate the restrictive assumption of burden tests of the same direction of effect of all rare variants on the trait within the same functional unit or genomic region, but require permutation procedures to assess statistical significance, which may not be computationally feasible for genome-wide analyses in large samples. To overcome this limitation, dispersion tests consider a more general function *f*(.), given by

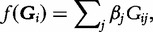



where *β_j_* denotes the effect of each copy of the minor allele at the *j*th rare variant. Of course, for rare variants, the allelic effects, **β**, cannot be reliably estimated. Consequently, the sequence kernel association test (SKAT) [26] makes the assumption that 
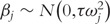
, where, as before, the weights *ω_j_* denote the masking scheme, and now *τ* is an unknown variance component parameter. Under the null hypothesis of no association between the trait and rare variation in the functional unit or genomic region, *β_j_* = 0 for all *j*, and is thus equivalent to *τ* = 0. SKAT uses a variance-component score test, given by

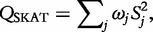



where

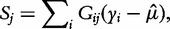



and 
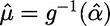
 is the expected trait value under the null hypothesis of no association. In the special case of a dichotomous phenotype with no covariates, SKAT is equivalent to the C-alpha test [27]. *Q*_SKAT_ follows a weighted sum of 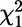 distributions under the null hypothesis, the significance of which can be determined analytically, without the need for permutations.

Burden and dispersion tests have been designed to test for association of rare variants in the same functional unit or genomic regions under different models of the effect of minor alleles on a complex trait, in particular, their direction of effect on phenotype. In an attempt to develop an approach that would be applicable across a wider range of association models, Lee *et al.* [28] proposed a linear combination of burden and dispersion score tests, constructed within the SKAT analysis framework. More specifically,

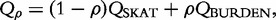



where

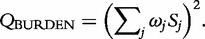



For a fixed mixture parameter, *ρ*, the test statistic *Q_ρ_* follows a weighted sum of 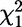 distributions under the null hypothesis of no association. Alternatively, *ρ* can be treated as an unknown nuisance parameter, and a data-driven procedure, SKAT-O, used to evaluate significance, without the need for computationally intensive permutations. A similar framework, combining a variance component and generalised burden test as independent score statistics, using Fisher’s or Tippett’s procedures, has been implemented in the Mixed effects Score Test (MiST) [29].

### Adaptive clustering methods

An alternative approach to allow for rare genetic variants within a functional unit or genomic region to have different direction and/or magnitude of effects on a complex trait is to make use of a kernel-based adaptive cluster (KBAC) [30], which categorises individuals according to ‘genotype groups’. In general, there are 3*^J^* possible genotype groups across a set of *J* variants. However, for rare variants, most of these possible genotype groups will not be seen because of low MAF, and, instead, we observe only *M* + 1 patterns, denoted *P*_0_, *P*_1_, … , *P_M_*, where *P_0_* represents a pattern of common homozygotes only. The advantage of KBAC is that the genotype patterns encompass a wide range of possible models of association; for example, allowing for interactions between rare variants that cannot be easily incorporated with simple linear functions, *f*(.). For KBAC, a kernel *K_m_* is defined for each pattern *P_m_* of genotypes. Consequently, the function *f*(.) can be expressed as *f*(***G****_i_*) = *γK_m_*, where *P_m_* is the pattern of genotypes carried by the *i*th individual, and a score test of the null hypothesis of no association of the trait with rare variants in the functional unit or genomic region, *γ* = 0, constructed for the specified kernel.

For dichotomous disease phenotypes, a hyper-geometric kernel is appropriate, and it is given by

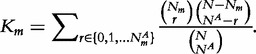

In this expression, *N* is the total number of individuals in the study, of which *N_m_* carry genotype pattern *P_m_* across rare variants in the functional unit or genomic region, and *N^A^* and 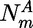 denote the same quantities, respectively, in cases. For this kernel, in the absence of covariates, the KBAC score test is given by

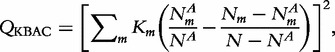



with significance assessed via permutation.

## POWER OF RARE VARIANT METHODS TO DETECT ASSOCIATION WITH COMPLEX TRAITS

As described above, there is a huge range of methodology available to detect association of complex traits with rare genetic variation in the same functional unit or genomic region. The majority of methods have been developed in the flexible GLM framework, but impose different underlying models of association that would be expected to be most powerful when the specific modelling assumptions are correct. For example, we might expect that the burden tests will be most powerful when all rare variants, after application of an appropriate masking scheme, have the same direction of effect on the complex trait. However, dispersion tests would be expected to be more robust to neutral variants, or to those with opposite directions of effect on the trait. Consequently, it seems unlikely that there will be a single ‘uniformly most powerful’ rare variant association test over all possible underlying genetic architectures.

Ladouceur *et al.* provide one of the most comprehensive evaluations of rare variant methodology to date [31]. They assess the comparative power of several burden tests, as well as SKAT and an adaptive clustering method (inspired by KBAC). They employ Sanger sequencing data from ∼2,000 individuals at seven genes, and simulate continuous traits over a range of genetic models spanning different hypotheses for the effects of rare genetic variation in the genes. They also investigate the performance of rare variant methods for dichotomous traits by using 500 cases and 500 controls selected from the extremes of the distribution. As seen previously [32], the power across tests was found to be affected by the proportion of causal variants in a gene, as well as their effect sizes. While the power of tests on continuous traits increased monotonically with larger effect sizes, tests on dichotomous traits seemed to be less affected. The power of collapsing tests increased more sharply as the number of causal variants increased. The VT method outperformed alternatives in scenarios where rarer variants had stronger effects, but only for continuous phenotypes. SKAT was found to be more powerful than alternatives when mixtures of deleterious and protective variants were driving the association, as expected. SKAT was also the most powerful approach when a combination of common and rare variants was driving the association.

Given that burden and dispersion tests appear to have differential advantages, tests combining the two approaches seem like an attractive alternative. Indeed, both SKAT-O and MiST have been reported as performing well under a range of phenotypes with varying causal to total variant distributions, irrespective of their direction of effect [28, 29]. However, these methods are still to be subjected to independent evaluation. A comparison of rare variant methods on larger (>1000) sample sizes would also be particularly informative, since most comparative studies to date [28, 29, 31, 32] have been conducted on smaller sample sizes than ongoing sequencing efforts.

The power of rare variant association methodology is also likely to vary according to the technology used to assay genetic variation. Magi *et al.* [16] undertook simulations to evaluate the relative performance of different design strategies to identify association of a quantitative trait with rare variants in a 50 kb gene using GRANVIL, including: (i) re-sequencing; (ii) genotyping of all variants present in a reference panel from the same population; and (iii) imputation of a GWAS scaffold of primarily common variants up to the reference panel using IMPUTEv2 [33]. They considered a model in which the expected trait value of an individual was increased by the presence of a minor allele at any causal variant in the gene. The trait association model was then parameterised in terms of: (i) the maximum MAF of any causal variant in the gene; (ii) the total MAF of all causal variants in the gene; and (iii) their joint contribution to the trait variance. They also considered a range of sizes for the reference panel, varying from 150 to 4000 individuals, reflecting current and future efforts from the 1000 Genomes Project [13] and the UK10K Project (www.uk10k.org).

As expected, the most powerful strategy to detecting rare variant association was through re-sequencing, which, in the absence of calling and genotyping errors, provides a complete catalogue of genetic variation in the gene. However, a strategy of genotyping all rare variants present in a large, population-matched reference panel, results in a relatively small reduction in power. Rare variants not captured by the reference panel (such as private mutations or those of very low frequency) are less likely to have a major impact on the trait under their simulation model, and thus, would not be expected to lead to a dramatic reduction in power. In the same way, imputation of a GWAS scaffold up to a large, population-matched reference panel also retains much of the power of the re-sequencing strategy. Larger reference panels provide more comprehensive coverage of a rare variation in the gene, and higher quality imputation, allowing recovery of genotypes at variants with MAF as low as 0.3% [34]. However, imputation of variation of lower MAF remains a considerable challenge, and it is not clear that the quality metrics used for common SNPs will be sufficient for removing poorly performing rare variants from downstream association analyses [35]. For this reason, imputation can never replace the ‘gold standard approach’ to assaying rare genetic variation through re-sequencing, although it currently provides a financially feasible, complimentary strategy to detecting association with complex traits in the required large sample sizes at a fraction of the cost.

## DISCUSSION

Statistical methodology for the analysis of rare genetic variation in the next generation of GWAS has been primarily developed in a flexible GLM framework, which can be applied to directly assayed or imputed genotype data and to quantitative traits or dichotomous disease phenotypes. The majority of statistical methods can be classified as burden tests, which assume the same direction of effect on the trait of all rare variants, dispersion tests, which allow for deviations from this unidirectional assumption, or a combination of the two approaches. The relative utility and power of these approaches depend on: (i) the computational burden (e.g. the need for permutations to evaluate statistical significance); (ii) the reliability of annotation (e.g. identification of coding variation that is more likely to have functional consequences); and (iii) the alignment of modelling assumptions with the underlying genetic architecture of the trait (e.g. robustness to neutral variants and an assumption of all causal alleles having the same magnitude and direction of effect). Simulations highlight that there is no uniformly most powerful approach but that methods that combine burden and dispersion tests are relatively robust to various underlying genetic architectures.

Until recently, rare variant association studies have been limited to candidate genes (functional or positional in GWAS loci) because of the expense of re-sequencing in large sample sizes. Despite these constraints, confirmed rare variant associations include: (i) plasma lipid concentrations with *ABCA1*, *APOA1*, *LCAT*, *NPC1L1* and *ANGPTL4* [36–38]; (ii) body mass index with monogenic obesity-related genes [39]; (iii) blood pressure with renal salt handling genes [40]; (iv) hypertriglyceridemia with lipoprotein lipase [41]; (v) inflammatory bowel disease with *NOD2* [42]; and (vi) type 2 diabetes with *MTNR1B* [43]. However, with recent improvements in the throughput and efficiency of re-sequencing technologies and advances in statistical methodology to allow imputation of existing GWAS scaffolds up to high-density reference panels, genotypes at rare genetic variants are becoming increasingly interrogated in the sample sizes required for complex human traits. Consequently, genome- and exome-wide analyses of rare genetic variation have identified novel genes implicated in high-density lipoprotein cholesterol [44], insulin processing and secretion [45], and type 2 diabetes [46].

Despite these success stories, further methodological development to maximise the potential of next-generation GWAS to identify rare variant associations with complex human traits is required. Improved functional annotation and a better understanding of the role of non-coding regulatory variation (e.g., through the ENCODE Project Consortium [47]) will inform study design and define powerful weighting schemes for rare variant analyses. Methodology to enable meta-analysis of rare variant association tests [48–50], by combining summary statistics across GWAS, would be expected to increase power but may be complicated by the observation that lower-frequency variation is more likely to be population-specific and, thus, may not be shared between studies, particularly in a trans-ethnic context. Nevertheless, with continued methodological development and increased availability of next-generation GWAS of rare genetic variation, the coming years offer an exciting opportunity to discover novel genes implicated in complex human traits and an improved understanding of the genetic architecture and pathophysiology of human disease, with the ultimate goal of developing effective clinical intervention, resulting in improved public health.

**Key points**

- There has been recent speculation that rare genetic variants, typically defined to have a minor allele frequency of less than 1%, might account for much of the missing heritability of complex human traits.
- Traditional statistical methods for the analysis of common SNPs in genome-wide association studies are underpowered for rare variants.
- There has been an exciting period of research and development into the methodology for the analysis of rare genetic variants by considering their joint effects on complex traits within the same functional unit or genomic region.

## FUNDING

APM acknowledges financial support from the Wellcome Trust (grant numbers WT098017 and WT090532)
